# Women empowerment and practices regarding use of dual protection among family planning clients in urban Zimbabwe

**DOI:** 10.11604/pamj.2014.17.300.3282

**Published:** 2014-04-20

**Authors:** Jesca Mutowo, Christine Mary Kasu, Esther Mufunda

**Affiliations:** 1 Department of Health Sciences, Zimbabwe Open University, Harare, Zimbabwe; 2 Department of Health Sciences, University of Zimbabwe, Harare, Zimbabwe

**Keywords:** Dual protection, family planning, women empowerment, condom

## Abstract

**Introduction:**

Gender related vulnerability may increase women's susceptibility to HIV infection and unintended pregnancy. The purpose of the study was to examine the relationship between women empowerment and practices regarding use of dual protection.

**Methods:**

A non-experimental descriptive correlational study design was conducted using systematic sampling method to recruit eighty women aged 18-49 years at an urban clinic in Zimbabwe. Data was collected using a structured interview schedule and was analysed and presented using descriptive and inferential statistics.

**Results:**

A weak positive significant correlation existed between women empowerment and use of dual protection (r= .242, p=0.03). Findings demonstrated that as women empowerment levels increase practices regarding use of dual protection also increase. The coefficient of determination, R2=.0.058, b=0.293, indicated that the total amount of variation in utilization of dual protection explained by level of women empowerment was 5.8%. The major finding was that use of dual protection was very low (3.8%) and 67.5% had low levels of practices regarding use of dual protection. Additionally, 85.0% were not confident of using the female condom.

**Conclusion:**

Gender inequality within sexual relations was associated with low levels of practices regarding use of dual protection. The study provided evidence for the need for a proactive integrated approach to empower women so that they could negotiate for safer sex practices. To increase female condom utilization, manufacturers need to redesign the female condom so that it becomes user friendly. Health personnel need to involve men for any health reproductive program to succeed.

## Introduction

HIV and AIDS is affecting an increasing number of women. Sexual and reproductive health rights emphasize equitable access to prevention and care. Unfortunately due to social, economic and political inequities between men and women there is unequal access to prevention, education and care [1]. The rights of individuals and couples to enjoy a healthy sexual life includes the prevention of unwanted pregnancy and sexually transmitted infections (STIs), including HIV [2].

HIV-related diseases are the leading causes of death among women aged between 15-49 years of age worldwide [3]. Nearly 60% of the world's 15.5 million HIV infected women live in Sub-Saharan Africa [4]. Rates of unintended pregnancy, HIV and other sexually transmitted infections are also high and contribute to poor health outcomes [5]. HIV prevalence among Zimbabwean women aged 15-49 is 18% [6] and these are current or potential users of contraceptive methods. Increased rates of unintended pregnancies and HIV infection call for use of dual protection, which combines the condom (either male or female) plus a hormonal method of contraception or permanent (sterilization) or long acting (intra-uterine device or implant) or natural methods. When used correctly and consistently, condoms alone can serve a dual purpose of protecting against unintended pregnancy and HIV and AIDS infection [7].

Generally, motherhood is considered a feminine ideal, hence using barrier methods as a safer sex option presents a significant challenge to many married women who plan to have children. Because of this perception, coupled with unequal power relations and economic disparities, women are unable to insist on safe sexual practices, especially dual protection. Poverty increases vulnerability to unsafe sexual behaviours and practices and it also decreases access to sexual and reproductive health information and services [1] which might expose women to unplanned pregnancies, unsafe abortions and transmission of HIV and AIDS. Women need to be economically empowered so that they can be assertive and be able to negotiate safe and responsible sexual practices, including the use of condoms. About two thirds of the illiterate adults in the world are females [4] and this compromises their decision making power in matters that affect their sexual and reproductive health. Education empowerment enables women to make informed choices that help prevent HIV infection and unwanted pregnancy.

The Millennium Development Goal 3 addresses gender equality and women empowerment in an effort to encourage women to participate in matters concerning their lives, including making choices in sexual and reproductive health matters. Therefore, women need to be empowered in sexual and reproductive health issues to contain and reverse the AIDS epidemic [8]. A study done in South Africa revealed a low level of dual method use of 16% [9], while another done in Zimbabwe reported dual protection use of 38% [10]. This figure is worrying for a country with a high HIV prevalence of 15% [6]. The contraceptive prevalence rate for Zimbabwe is at 60.2% [3]. However, because of the separation of services between family planning and other curative services, many women in family planning clinics are receiving contraceptive services with little attention to their need for dual protection [11].

The female condom was developed as an alternative to male condom as a means to empower women so that they could have control over their own protection against STIs, HIV infection and unintended pregnancy [12]. Unfortunately, female condoms are less promoted and not easily available than male condoms [13]. In Cambodia, women demonstrated a high awareness of the benefits of condom use, but 75% were unaware of the existence of the female condom [14]. Poor and marginalised women suffer the greater burden of sexual and reproductive ill health such as unwanted pregnancy, unsafe abortions, HIV and AIDS infection. These consequently lead to increased morbidity and mortality. There is limited literature on dual protection [7] and a few studies have looked on how individuals perceive their own risk and the need for dual protection use [13].

Therefore, the purpose of the study was to examine the relationship between women empowerment and practices regarding use of dual protection among women aged 18 to 49 years at a Family Health Services Clinic in Zimbabwe.

## Methods

### Design

A non-experimental descriptive correlational study design was used. The design enabled the investigators to gather information and make inferences about possible relationship between women empowerment and practices regarding use of dual protection.

### Participants

The study sample consisted of 80 women on family planning methods who were seeking services for their under five children during the period of data collection. The participants were recruited using systematic sampling method. The inclusion criteria were women who reported using a contraceptive method, either modern or natural, during the month of their interview or at their last intercourse in the past three months, women aged between 18 to 49 years and fluent in Shona or/ and English.

### Data Collection

A structured interview schedule was used to collect data on women empowerment, practices regarding use of dual protection and demographic variables. Face to face interviews were conducted in a private room to control extraneous variables and each interview lasted 20-25 minutes. The interviews were done between 8am and 1pm during weekdays in order to maximise on the increase in the flow of clients in the morning.

### Data collection instrument

A structured interview schedule was used to elicit data from the participants. Face to face interviews were conducted to provide the interviewer with the opportunity to elicit and probe for more information when participants made non-verbal cues, such as facial or body expressions and change of tone of voice. The interview guide was translated from English to Shona. Content validity of the instrument was ensured by rigorous effort in designing the questions and was assessed by a panel of experts at the clinic. Reliability of the instrument was tested using Cronbach's Alpha coefficient and it ranged between 0.7322 and 0.7350. A pilot study was conducted on 5 participants who met the inclusion criteria but were not included in the final sample. The revision was on language usage that was familiar to family planning women.

The dependent variable was practices regarding use of dual protection and was addressed by the Practices Regarding Use of Dual Protection Interview Schedule (Appendix B, Section B). It had 25 questions with one correct answer among the given choices, except questions 8, 9 and 15, where participants gave more than one answer. The interview also elicited information on perceived susceptibility to HIV infection and what motivated the participants to use dual protection. The last two questions were descriptive in nature and sought to find out participants’ opinion, and were not scored. All dual protection questions in the interview schedule were given scores on a continues scale up to 36. Practices regarding use of dual protection were classified into three categories; low (≤18), moderate (19-28) and high (≥29).

The independent variable was women empowerment and was addressed by the Women Empowerment Interview Schedule (Appendix B, Section C). It comprised 21 questions each with one correct answer among the given choices. Questions included information on educational level, employment status, participation in family decision making, control of economic resources, ownership of resources, intimate partner violence, sexual and reproductive health rights and care, and woman's status in the family. Women empowerment factors on the practices regarding use of dual protection were scored on a continuous scale with a maximum of 34. These were categorised to level of empowerment on practices regarding use of dual protection to: lowly empowered (0-17), moderately empowered (18-24) and highly empowered (≥25). The socio-demographic data included age, marital status, religion and place of residence.

### Ethical consideration

The study was approved by the Joint Research Ethics Committee (JREC), the Medical Research Council of Zimbabwe (MRCZ) and the Director of Health Services, City of Harare. Written consent was obtained from the subjects showing their willingness to participate in the study.

### Data Analysis

Statistical and descriptive analysis involved the determination of the mean, mode, medium, frequencies and standard deviation (SD). Pearson's correlation matrix was used to determine the relationship between women empowerment and practices regarding use of dual protection among women aged between 18 to 49 years. A code book was used to identify and define each variable in the study. The Statistical Package for the Social Sciences software was used to analyse the results. The test was performed with a significance level of p= 0.05.

## Results

### Sample Demographics

Table 1, summarises the demographic profile of the participants. The age ranged between 18 to 46 years (mean 27 years, SD 6.7 years).

**Table 1:** Sample Demographics

Variable	Frequency (n)	Percentage (%)
**Age (years)**		
18-25	34	42.5
26-35	35	43.8
36-49	11	13.7
**Marital status**		
Single	4	5.0
Married	72	90.0
Divorced	1	1.2
Widowed	0	0.0
Separated	3	3.8
**Religion**		
Pentecostal	38	47.5
Catholic	5	6.2
Protestant	9	11.3
Other	28	35.0
**Residents**		
Urban	75	93.8
Rural	4	5.0
Mining	1	1.2
N= (80)		

### Practices Regarding Use of Dual Protection

**Figure 1:**
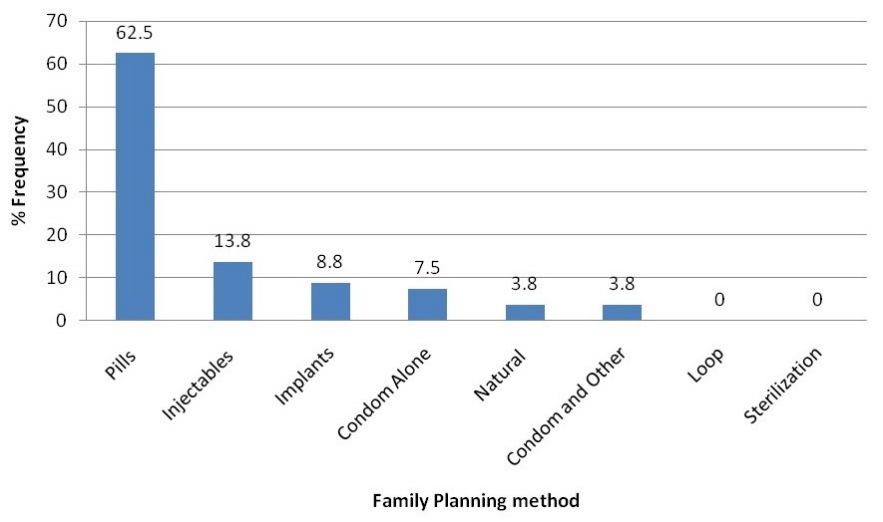
Family Planning method use

Figure 1 shows family planning methods used by the participants. Most of the participants used the pill; very few used a natural method (lactation amenorrhea) and condoms with another family planning method.

Table 2 shows that of those who reported using condoms with their partners most had received instructions on condom use from health personnel. Most of the participants reported that male condoms where user friendly. Very few were confident of using the female condom compared to the male condom. Overall the participants acknowledged that condoms were effective in preventing pregnancies and STI including HIV. A small percentage (1.2%) had high level of dual protection practices.

**Table 2:** Practices regarding use of dual protection

Variable	Frequency (n)	Percentage (%)
**Received instruction on condom use**		
Yes	48	60
Confident in using male condom		
Yes	49	61.2
Confident in using female condom		
Yes	12	15.0
**Condom effectiveness in preventing pregnancy**		
Effective	49	61.2
Fairly effective	26	32.5
**Condom effectiveness in preventing STI including HIV**		
Effective	44	55.0
Fairly effective	28	35.0
**Condom use a sign of mistrust**		
Yes	28	35.0
**Level of dual protection Practice**		
High	1	1.2
N=(80)		

### Women Empowerment

The age range at first marriage was 15 to 28 years. Fifty percent of the participants were younger than their partners/husbands with an age difference of 5 years or less, while (6.2%) were more than 10 years younger than their partners/husbands. The majority (91.2%) of the participants were educated up to secondary level. Most (68%) of the participants were unemployed and none of the participants contributed significantly to the total family income. Most of the participants were not empowered economically, socially and culturally (Table 3). Fifty-four (67.5%) of the subjects had a low level of empowerment as they scored <17. The rest were moderately empowered 25 (31.3%) to highly empowered 1 (1.2%) and scored between 18-24 and >25 respectively.

**Table 3:** Women empowerment factors

Variable	Frequency (n)	Percentage (%)
**Who makes economic decisions?**		
Partner	39	48.8
Self	12	15.0
Both	29	36.2
**Owns assets**		
No	53	66.2
**Ability to refuse unprotected sex if partner had a sexually transmitted infection**		
Yes	69	86.2
**Have a say on Family Planning**		
Yes	60	75.0
**Easiness in negotiating safe sex**		
Easy	29	36.2
Fairly easy	23	28.8
Not easy	28	35.0
**Culture supportive of women assertiveness on sexual matters**		
No	71	88.8
**Would justify partner/husband beating you if you refuse sex**		
No	75	93.8
N=80		

### Study correlation coefficient measurements of women empowerment and practices regarding use of dual protection

Table 4 shows the Pearson correlation coefficient analysis for examining the relationship between women empowerment practices regarding use of dual protection and their p values. A positive significant correlation existed (r= .242, sig 5%, p=.03). at (sig 5%).

**Table 4:** Pearson's correlation matrix of practices regarding use of dual protection and women empowerment

		Y
		1.00
**X**		.242*
*p<.05	**p<.01	***p<.001
Y= practices regarding use of dual protection; X= women empowerment		

Factors that were significantly correlated with women empowerment in use of dual protection were age difference at marriage, level of education, employment status, income, economic decisions, ability to refuse sex if partner had a sexually transmitted infection, having a say in family planning matters, awareness of sexual and reproductive health rights, protection of reproductive health rights, ability to make health decisions and easiness of negotiating for safe sex.

The Regression analysis coefficient (b=0.293) showed a weak positive relationship showing its statistically significant effect on the dependent variable. The regression showed that empowerment level was a significant predictor of practices regarding use of dual protection. The total amount of variation in practices regarding use of dual protection explained by level of women empowerment was 5.8%.

## Discussion

The overall dual protection use and practices regarding use of dual protection in this sample was very low as evidenced by only 3, 8% participants using effective dual protection. This prevalence figure is lower than populations studied in South Africa [9] and in Zimbabwe [10] in which 16% and 38% used dual protection respectively. The majority of the participants in this study were married. The very low prevalence of dual protection use within marriage in this study is an important indicator of how difficult it is to use condoms within marriage. This assumption is supported by [9, 15, 16] who also assert that condom use within marriage is uncommon and is highly stigmatized. These results show that there is a high HIV risk in married women. The results also showed that the majority of the participants were using hormonal methods of contraception instead of natural methods. The results concur with results from a study in South Africa [17] in which most of the participants used hormonal methods of contraception. Exposure to both electronic and print media on health related education and information was likely to have had a positive influence on the participants as most of them lived in urban areas.

Participants in this study preferred the male condom. This is in contrast to a study done in Zimbabwe [18], in which most male and female participants preferred female condom to male condom. In terms of empowerment levels, participants in this study had reduced empowerment levels as they preferred the male condom which they had little or no control over its use and application. Preference for male condom could also be due to the fact that male condom has been in existence since time immemorial and change to female condom might be difficult to implement. These results are cause for concern for policy makers as the female condom was developed as an alternative to male condom as a strategy to empower women so that they could have control over their own protection against STIs, HIV infection and unintended pregnancy thereby reducing mortality and morbidity.

Though most of the participants had received instructions on condom use this was not converted to an increase in condom use. Fifty-four (67.5%) reported that male condoms were user friendly and only 15 (18.7%) said female condoms were user friendly. The results show that the male condoms were easy to use for many couples hence increased preference, which hopefully would subsequently lead to utilization. There was an increased perceived efficacy of condom as a protective measure against pregnancy and STI including HIV. These results are similar to those from a study done in KwaZulu Natal [19], in which condoms were widely viewed as an effective method of preventing HIV infection. Therefore low usage of condom in this study is not due to perception of effectiveness but rather attitudes towards it and probably perceived reduced self efficacy. A low proportion of the participants reported that they consistently (always) used condoms with their partners/ husbands. The results were consistent with study findings from KwaZulu Natal [19] in which only 15% men and 18% women reported consistent use of condoms. The results of this study show that some married couples are willing to use condoms at least some of the time if they perceive there is a risk of infection or getting pregnant. Hence there is need to target these women in HIV prevention programs because condoms can be used in some marriages. According to [16], couples who communicate openly about safer sex practices are more likely to reduce behavioural risks than couples who do not discuss. In this study, very few participants had discussed dual protection with partner/ husband. With the identification of discordant couples there is need for midwives to reinforce the need for couple counselling and testing and greater condom use in stable relationships. In order for any interventions to achieve its goal there is need to involve men, so that they can also take responsibility.

Contrary to results from a study carried out in South Africa [15], where participants’ perceived risk of infection was predictor to condom use, participants in this study's perceived risk of STI including HIV infection from partner/ husband and access to condoms did not emerge as the predictor of condom use. It is important to note that the majority of participants in this study had never suffered from a sexually transmitted infection. These results may indicate positive behavioural changes in light of the HIV and AIDS epidemic.

The majority of the participants were unemployed. Therefore they were economically dependent. This may negatively affect their ability to make independent decisions including safer sex practices. Poverty increases incidences of sexual and reproductive ill health, such as unsafe abortions and exposure to HIV infection leading to maternal morbidity and mortality. Some form of assertiveness was shown in the majority (86.2%) of the participants as they were able to refuse unprotected sex if their partner/ husband had a sexually transmitted infection, which shows some level of empowerment. Women need to be empowered with negotiation skills to cater for those who fail to negotiate effectively.

Cultural practices such as subservient role in sexual relations are not favourable to women empowerment in terms of use of dual protection. The majority 71 (88.8%) reported that their culture did not support assertiveness in women on sexual matters. These results echo the sentiments expressed by [20], who noted that women are expected to be passive and assertiveness on sexual issues would be viewed unfeminine. This means that traditional factors should be addressed as they have a strong effect on women's autonomy and ability to negotiate safe sex practices. The majority had low empowerment levels and this could be explained by the influence of culture on sexual and reproductive issues, which detects what is expected of women.

The results revealed that family planning clients in this study were not empowered economically, socially and culturally. This lack of empowerment might have contributed to reduced practices regarding use of dual protection exposing the women to unintended pregnancy and transmission of STI including HIV infection.

The results also identify gaps in reproductive health programmes that do not involve men. There is need for midwifery practice to advocate for male involvement in sexual and reproductive health programmes in order to ensure male support, especially in dual protection programmes.

The research findings have shown that any 5.8% change or variation in practices regarding use of dual protection was explained by women empowerment levels. This implies that poor practices and utilization of dual protection may be due to other factors other than lack of empowerment.

## Conclusion

Although the use of modern methods of family planning has been embraced by many the uses of dual protection and practices regarding use of dual protection was very low among the study sample. The study provided evidence for the need for a proactive integrated approach to empower women economically, socially and culturally so that they could be able to negotiate for safer sex practices. Effective intervention programmes need to encourage men and women to talk openly about use of dual protection. There is need for a cultural paradigm shift that transforms relations from male domination to mutual ones where discussions about sexual issues are encouraged and are not construed to be a sign of infidelity. Nurses and midwives could conduct community awareness campaigns especially at the work place emphasizing the effectiveness of simultaneous use of family planning methods and condoms to combat the spread of HIV and reduce incidences of unintended pregnancy. Intensive health education campaigns concentrating on demonstrations and return demonstrations on female condom application should be carried out in order to improve skills on proper use and build confidence in the use of the female condom. Manufacturers of the female condom may need to redesign it so that it becomes user friendly to increase its acceptability. Further research on a broader scale need to be conducted in other settings to investigate other factors affecting practices regarding use of dual protection since this study indicated that only 29% change in utilization was explained by change in women empowerment, so that any gaps in practice can be identified and addressed so as to improve the body of knowledge.

The major limitations of the study were that although the content validity of the instrument was rigorously assessed by clinical experts, it was used for the first time in this study and it did not undergo psychometric test. The sample size was small and this reduces generalizability of the findings to the entire population of Zimbabwe. Data was obtained through self-reports given by the subjects and this may have introduced bias as socially desirable responses may have been given.
